# Direct TaqMan assay for the detection and genotyping of bovine viral diarrhea virus types 1 and 2

**DOI:** 10.1007/s00705-024-06207-z

**Published:** 2024-12-12

**Authors:** Shakir Ullah, Kosuke Notsu, Akatsuki Saito, Tamaki Okabayashi, Hirohisa Mekata, Norikazu Isoda, Satoshi Sekiguchi

**Affiliations:** 1 Graduate School of Medicine and Veterinary Medicine, University of Miyazaki, Miyazaki, 889-1692 Japan; 2 Livestock and Dairy Development Department Balochistan, Quetta, 87300 Pakistan; 3 Center for Animal Disease Control, University of Miyazaki, Miyazaki, 889-2192 Japan; 4 Department of Veterinary Science, Faculty of Agriculture, University of Miyazaki, 1-1 Gakuen-Kibana-Nishi, Miyazaki, 889-2192 Japan; 5 Laboratory of Microbiology, Department of Disease Control, Faculty of Veterinary Medicine, Hokkaido University, Sapporo, Hokkaido 060-0818 Japan; 6 One Health Research Center, Hokkaido University, Sapporo, Hokkaido 060-0818 Japan; 7 International Collaboration Unit, International Institute for Zoonosis Control, Hokkaido University, Sapporo, Hokkaido 001-0020 Japan; 8 Institute for Vaccine Research and Development (HU-IVReD), Hokkaido University, Sapporo, Hokkaido 001-0021 Japan

## Abstract

Bovine viral diarrhea (BVD), caused by bovine viral diarrhea virus (BVDV), has a significant economic impact on affected farms worldwide. For effective disease control, it is crucial to select an appropriate vaccine based on the specific genotype of BVDV. Therefore, developing a rapid and reliable assay to detect and genotype BVDV is imperative for controlling the spread of disease. In this study, we developed a TaqMan assay to detect and genotype BVDV types 1 and 2 directly in bovine serum without extraction of RNA. The direct BVDV TaqMan assay effectively detected both BVDV1 and BVDV2 with confirmed specificity and showed no cross-reactivity with any of the other viruses tested, including bovine respiratory syncytial virus, bovine coronavirus, Akabane virus, bovine herpesvirus 1, bovine parainfluenza virus 3, bovine immunodeficiency virus, and bovine leukemia virus. The assay could detect the virus in serum samples with a titer as low as 10^2^ TCID_50_/mL in two out of three trials for BVDV1 and all three trials for BVDV2, indicating that its sensitivity is equivalent to that of virus isolation. Our findings represent a significant advancement in BVDV detection and typing directly from bovine serum.

**Supplementary Information:**

The online version contains supplementary material available at 10.1007/s00705-024-06207-z.

## Introduction

Bovine viral diarrhea virus (BVDV), the causative agent of bovine viral diarrhea, belongs to the genus *Pestivirus* of the family *Flaviviridae*. In addition to cattle, BVDV affects other species such as sheep, goats, camels, and swine. Three genotypes of BVDV have been identified worldwide: BVDV1, BVDV2, and BVDV3 (HoBi-like pestivirus), with BVDV1 being the most prevalent [1]. Epidemiological studies have shown that BVDV2 is present in South America, Europe, the USA, and Japan [1, 2], whereas BVDV3 has a more limited distribution, including South America, Europe, and Asia [3]. BVDV has a severe economic impact on the cattle industry due to respiratory infections, decreased milk production, abortion of fetuses, the birth of weak and persistently infected (PI) calves, and long-term infections [4]. The PI animals serve as viral reservoirs within herds and continually shed the virus in large amounts [5]. The presence of these reservoir animals raises the possibility of the virus spreading throughout the herd and increasing the prevalence of the virus. Early diagnosis of BVDV is therefore crucial, with the main goal being to identify and remove BVDV PI cattle from herds. Effective diagnosis can support BVDV eradication programs and the economic growth of the cattle sector.

Currently, the diagnosis of BVDV infection relies on several laboratory techniques, including immunohistochemistry (IHC), capture enzyme-linked immunosorbent assay (ELISA), virus isolation (VI), RT-PCR, and real-time RT-PCR [6, 7]. Each laboratory technique has its strengths and limitations regarding the diagnosis of BVDV. For example, IHC and VI are time-consuming and are incapable of genotyping BVDV isolates. Capture ELISA techniques offer more-rapid results than VI, but colostral antibodies have been reported to interfere with the results when samples are taken from calves that are fed colostrum [8, 9]. Moreover, neither ELISA nor VI can be used to identify the infecting BVDV genotype. Importantly, vaccination against BVDV1 does not protect against BVDV2, and vice versa, because of their antigenic differences. It is therefore essential to discriminate between BVDV1 and BVDV2 in order to choose an effective vaccine [10].

Genetic diagnostic methods such as PCR, loop-mediated isothermal amplification (LAMP), and recombinase polymerase amplification (RPA) provide robust platforms to diagnose BVDV easily and rapidly. A reverse transcription quantitative PCR (RT-qPCR) described by Hoffmann et al. is a World Organisation for Animal Health (WOAH) reference assay that can detect BVDV in a one-step (combined RT and subsequent PCR) reaction regardless of its genotype [11]. While Letellier *et al*. and Mari *et al.* have reported successful detection and discrimination of BVDV genotypes using this assay [12, 13], the RT and PCR steps were performed separately, increasing the time and complexity of the method.

Recent advancements in diagnostic techniques have focused on detecting pathogens without RNA extraction. The direct detection of SARS-CoV-2 exemplifies this approach [14]. To date, there have been no reports of the diagnosis and genotyping of BVDV directly from serum. To address this, we developed a TaqMan-based RT-PCR method for the sensitive and specific detection and differentiation of BVDV1 and BVDV2 directly in the serum of BVDV-infected cattle without RNA extraction. This assay can reduce the time, cost, and workload required to diagnose BVDV infections accurately.

## Materials and methods

### Ethics statement

The study protocol was reviewed by the Cattle Ethics Committee of the Faculty of Agriculture at the University of Miyazaki, Japan.

### Primer and probe design

The complete genome sequences of reference strains of BVDV were obtained from the GenBank database (http://www.ncbi.nlm.nih.gov/Genbank/index.html) and aligned using Molecular Evolutionary Genetics Analysis version 11 (MEGA11) software [15]. The data included genome sequences of four strains of BVDV1a, five strains of BVDV1b, two strains of BVDV1h, one strain each of BVDV1c, BVDV1f, BVDV1k, and BVDV1q, six strains of BVDV2a, one strain of BVDV2b, and two strains of BVDV3 (Supplementary Table S1). Based on this alignment, we identified a BVDV subtype-specific conserved region covering the 3ʹ end of the 5ʹ UTR and the 5ʹ end of the N^pro^ gene. Common forward and reverse primers for BVDV1 and BVDV2 were designed manually, and two specific probes recognizing the region between the forward and reverse primers were also designed to detect BVDV1 and BVDV2. The specificity of the primers and probes was verified using Primer-Blast (https://www.ncbi.nlm.nih.gov/tools/primer-blast/). These oligonucleotides were purchased from Eurofins Genomics (Tokyo, Japan), and their sequences are shown in Table 1.

**Table 1:** Primers and probes used in the TaqMan assay

Reference strain		Gene	Position	Primer/probe name	Sequence (5’ to 3’)	Length	Reverse compliment
LC0006970 (KZ-91-CP)		5' UTR	315-335	BVDV_63F	TRYGRAYACAGCCTGATAGGG	83 bp	
LC0006970 (KZ-91-CP)		5' UTR-N^pro^	376-396	BVDV_63R	WCAACTCCATGTGCCATGTACAG		CTGTACATGGCACATGGAGTTGW
M31182(NADL)		5' UTR	359-373	BVDV1_P1	FAM-ACTRAAAATCTCTGC-MGB-Eclipse		
LC0006970 (KZ-91-CP)		5' UTR	336-353	BVDV2_P2	HEX-TGTAGCAGAGACCTGCTA-MGB-Eclipse		

### Assessment of primer performance using a SYBR Green assay

Our assay was evaluated using our newly designed primers and a commercial SYBR Green assay kit (iTaq Universal SYBR Green One-Step Kit, Bio-Rad, Hercules, CA, USA). The 20-µL reaction mixture consisted of 10 µL of iTaq Universal SYBR Green Master Mix (2×), 0.3 µL of iScript reverse transcriptase (Bio-Rad), 750 nM each primer, 0.02 µL of 50× Rox reference, 2 µL of serum template, and 6.88 µL of nuclease-free water. Amplification was carried out using the following program: reverse transcription at 50 °C for 10 min and initial activation at 95 °C for 1 min, followed by 40 cycles of denaturation at 95 °C for 15 s and annealing at 62 °C for 20 s. This assay was performed using a QuantStudio 5 Real-Time PCR System (Thermo Fisher Scientific, Waltham, MA, USA).

### One-step BVDV-genotyping TaqMan assay

In the final protocol for the one-step BVDV-genotyping TaqMan assay, the 20-µL reaction mixture contained 5 µL of PrimeTime One-Step 4× Broad-Range Master Mix (IDT, Coralville, IA, USA), 750 nM each primer, 150 nM probe for BVDV genotype 1 (probe 1), 450 nM probe for genotype 2 (probe 2), 0.02 µL of 50× Rox reference dye, 2 µL of RNA template or bovine serum, and 11.38 µL of nuclease-free water. Amplification was carried out using the following program: reverse transcription at 50 °C for 15 min and initial activation at 95 °C for 3 min, followed by 40 cycles of denaturation at 95 °C for 15 s and annealing at 62 °C for 20 s. The fluorescence channels for this assay were set as follows: channel 1, FAM; channel 2, HEX. This assay was performed on a QuantStudio 5 Real-Time PCR System. A result was considered positive if the ΔRn (change in fluorescence) was above the threshold range (between 0.1 and 0.2) based on automatic/manual criteria.

### Bovine serum samples and RNA extraction

Available serum samples that were obtained from PI cattle or uninfected cattle and stored at ˗80 °C at the University of Miyazaki were used to evaluate the one-step TaqMan assay. The sera from PI cattle were collected by veterinarians during active surveillance conducted from 2016 to 2020 in Kyushu, Japan. RNA was extracted from these samples using a MagMAX CORE Nucleic Acid Purification Kit (Thermo Fisher Scientific) according to the manufacturer’s protocol and an automated Kingfisher Duo Prime nucleic acid extraction system (Thermo Fisher Scientific). The extracted RNAs were stored at ˗80 °C until further use.

### Cross-reactivity of the assay with BVDV3

To test the cross-reactivity of the one-step BVDV-genotyping TaqMan assay with BVDV3, three artificially synthesized RNA fragments containing sequences from BVDV3 isolates were obtained from FASMAC Co. Inc. (Kanagawa, Japan). These RNA fragments corresponded to 103 nt of the 5’ UTR-N^pro^ region, covering the primer/probe region of three representative BVDV 3 strains: Italy-1/10-1 (accession no. HQ231763), Th/04_KhonKaen (accession no. FJ040215), and JS12/01 (accession no. JX469119). These sequences are shown in Supplementary Table S2.

The copy number of these RNA fragments was calculated using the following formula: Copy number = (6.02) × 10^23^ copies/mol × plasmid concentration [ng/µL] × 10^-9^ / (RNA length in nucleotides × 660g/mol). The RNA copy number was adjusted to 10^5,^ 10^4,^ 10^3^, 10^2^, 10^1^, and 10^0^ copies by serial dilution in water and tested using the one-step BVDV-genotyping TaqMan assay.

### Cross-reactivity of the assay with other pathogens

The cross-reactivity of the one-step BVDV-genotyping TaqMan assay was tested using other selected bovine viruses. Bovine herpesvirus 1 (BoHV-1) strain RLB106, bovine parainfluenza virus 3 (BPIV-3) strain RLB103, and bovine respiratory syncytial virus (BRSV) strain BRSV/375 were obtained from the commercial vaccine TS RLB 106 (Zoetis, Kalamazoo, MI, USA). Akabane virus (AKAV) strain TS-C2 was obtained from the commercial Akabane disease live vaccine (Nisseiken, Tokyo, Japan). Bovine coronavirus (BCoV) strain CS5 [16] and whole blood from bovine leukemia virus (BLV)-infected cattle [17] were provided by the University of Miyazaki. From these samples, nucleic acids were extracted using either MagDEA Dx SV RNA reagent (Precision System Science, Chiba, Japan) with the automated nucleic acid extraction system magLEAD 12gc (Precision System Science) or a MagMAX CORE Nucleic Acid Purification Kit with an automated Kingfisher Duo Prime nucleic acid extraction system. A plasmid (pBIV127) containing the whole genome sequence of bovine immunodeficiency virus (BIV) was obtained from Dr. Charles Wood through the NIH AIDS Reagent Program, Division of AIDS, NIAID, NIH [18]. A one-step BVDV-genotyping TaqMan assay was performed on the above samples.

### Comparison of the limit of detection (LOD) using isolated RNA with that of the World Organization of Animal Health (WOAH) reference assay

To determine the LOD using isolated RNA, the one-step BVDV-genotyping TaqMan assay was compared with the assay described by Hoffmann *et al*. [11, 19], which was performed using the following 25-µL reaction mixture: 12.5 µL of 2× Reaction Mix with ROX, 0.5 µL of SuperScript III RT Platinum Taq Mix (Thermo Fisher Scientific), 800 nM BVD 190-F forward primer and V326 reverse primer, 120 nM TQ-pesti Probe, 5 µL of RNA, and 4.67 µL of nuclease-free water. The amplification profile was as follows: reverse transcription at 48 °C for 10 min and initial activation at 95 °C for 10 min, followed by 45 cycles of denaturation at 95 °C for 15 s and annealing at 60 °C for 1 min.

Two RNA samples extracted from sera from PI cattle were used: one from an animal that was infected with BVDV1 strain M5-3/G1B/Cattle/Japan/2018 (accession no. OR674901) and one from an animal that was infected with strain BVDV2 M2-2/G2A/Cattle/Japan/2018 (accession no. OR674907). These RNAs were extracted using a MagMAX CORE Nucleic Acid Purification Kit on a Kingfisher Duo Prime System. The RNAs were serially diluted tenfold using nuclease-free water, and this dilution series was used for both the one-step BVDV-genotyping TaqMan assay and the reference assay, which were performed in triplicate.

### Comparison of the LOD using bovine serum with that of VI

To evaluate the LOD using serum, the direct one-step BVDV-genotyping TaqMan assay was compared with VI, which is the gold standard for virus detection and is the assay with the highest sensitivity. For this purpose, the MDBK-HS cell line was maintained in Minimum Essential Medium Eagle (MEME, Sigma-Aldrich), containing 10% horse serum. The BVDV-negative status of the MDBK-HS cell line was confirmed using the assay of Hoffmann et al. [11]. The MDBK-HS cell line was cultured in a 96-well tissue culture plate. Two sera from PI cattle were used: one from an animal that was infected with BVDV1 strain M6-1/G1B/Cattle/Japan/2018 (accession no. OR674904) and the other from an animal that was infected with BVDV2 strain M2-1/G2A/Cattle/Japan/2016 (accession no. OR674894). First, the viral titers of these samples were determined as follows: Tenfold serial dilutions of the samples were made in MEME containing 10% horse serum, and 50 µL of each dilution was used to inoculate cells in a 96-well tissue culture plate in quadruplicate. After 3-5 days, the supernatants were removed, and the plates were dried overnight at room temperature in a safety cabinet. An immunoperoxidase assay for the presence of BVDV antigen was performed as described previously [20]. In this assay, we used the commercial anti-pestivirus monoclonal antibody JCU/BVD/CF10 (Trop Bio Pty Ltd, Queensland, Australia) as the primary antibody. The 50% tissue culture infectious dose (TCID_50_) was determined using the Reed–Muench method [21]. The virus concentration in the serum samples was adjusted to 10^3^, 10^2^, 10^1^, and 10^0^ TCID_50_/mL by dilution in MEME containing 10% horse serum, and the diluted samples were then incubated with MDBK-HS cells in triplicate. A sample was considered positive by VI if there was at least one positive well in the immunoperoxidase assay at 3-5 days post-inoculation.

In parallel, the viral titer of the serum samples was adjusted to 10^3^, 10^2^, 10^1^, and 10^0^ TCID_50_/mL by dilution with a bovine serum that was confirmed to be BVDV negative (qPCR negative by the assay of Hoffmann et al.) and negative for anti-BVDV antibodies (neutralization antibody titer <2). Subsequently, the one-step BVDV-genotyping TaqMan assay was performed in triplicate with each dilution.

### Correlation between the Ct value in the direct TaqMan assay and the viral titer

To estimate the viral titer of BVDV-infected cattle based on the direct TaqMan assay Ct value, the correlation between the Ct value in the direct TaqMan assay and the viral titer was analyzed. First, two sera of PI cattle (BVDV1 strain M6-1/G1B/Cattle/Japan/2018 and BVDV2 strain M2-1/G2A/Cattle/Japan/2016 were inoculated onto MDBK-HS cells to propagate the viruses. After propagation, the supernatant was collected and the viral titer (TCID_50_) was determined as described above. Then, the viral titers of the samples were adjusted to 10^6^, 10^5^, 10^4^, 10^3^, and 10^2^ TCID_50_/mL by dilution in BVDV-negative bovine serum. The direct TaqMan assay was performed in triplicate with each dilution, and the resulting Ct values were plotted against the viral titer. Correlation analysis was performed using R software (version 4.3.2, https://www.r-project.org/).

### Reproducibility of the direct one-step TaqMan assay

To verify the reproducibility of the one-step TaqMan assay, the intra-assay repeatability was evaluated by testing BVDV-infected sera in triplicate. For this, we used data obtained in the experiments described above. The coefficient of variation was calculated by dividing the standard deviation of each test supernatant by its mean and multiplying by 100.

### Agreement between the one-step BVDV-genotyping TaqMan assay and phylogenetic analysis for BVDV genotyping

To determine the accuracy of BVDV genotyping using our one-step BVDV-genotyping TaqMan assay, we assessed the level of agreement between our assay and phylogenetic analysis by testing previously phylogenetically characterized sera of PI cattle infected with local isolates of BVDV. These isolates included one strain of BVDV1a, seven strains of BVDV1b, and four strains of BVDV2a.

## Results

### Performance of newly designed primers and probes for BVDV detection

**Fig. 1:**
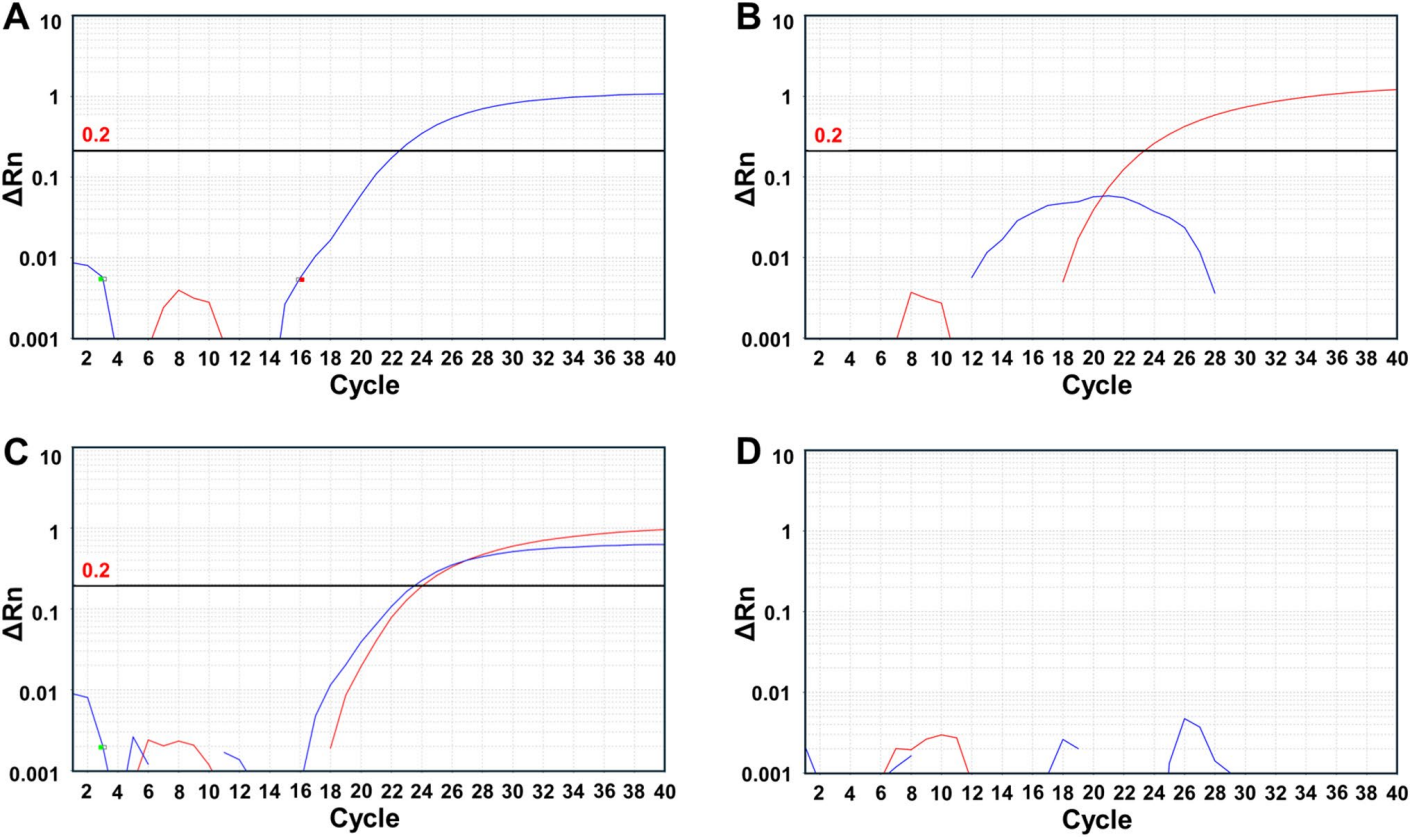
Performance of the TaqMan assay using isolated RNA. Panels (A-D) Amplification plots for the BVDV1 (FAM) and BVDV2 (HEX) channels are shown in blue and red, respectively. (A) BVDV1-RNA template. (B) BVDV2-RNA template. (C) Mixture of BVDV1 and BVDV2 RNA templates. (D) Extracted RNA from BVDV-negative cattle

**Fig. 2:**
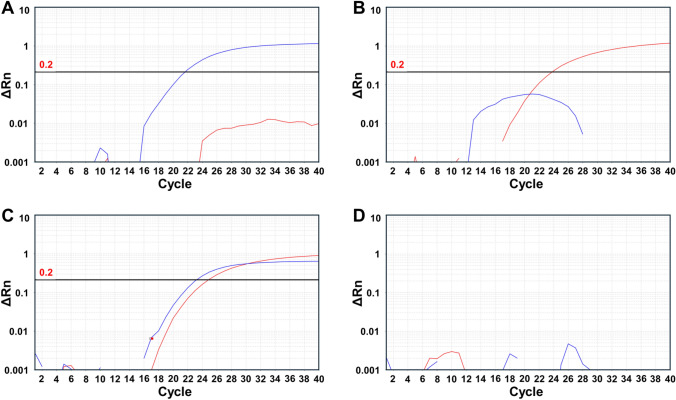
Performance of the direct TaqMan assay using supernatants from infected MDBK-HS cells without an RNA isolation step. (A-D) Amplification plots for the BVDV1 (FAM) and BVDV2 (HEX) channels are shown in blue and red, respectively. (A) Supernatant from BVDV1-infected cells. (B) Supernatant from BVDV2-infected cells. (C) Mixture of supernatants from BVDV1- and BVDV2-infected cells. (D) BVDV-negative cell supernatant

To assess the performance of the new primers and probes in the one-step BVDV TaqMan assay, we first tested extracted RNA obtained from the supernatant of MDBK-HS cells infected with BVDV1 strain M6-1/G1B/Cattle/Japan/2018 or BVDV2 strain M2-1/G2A/Cattle/Japan/2016. As shown in Fig. 1, the one-step BVDV TaqMan assay successfully detected RNA from BVDV1 (Fig. 1A), BVDV2 (Fig. 1B), and a mixture of BVDV1 and BVDV2 (Fig. 1C). A sample from uninfected cells gave a negative result (Fig. 1D). Next, the supernatants were tested as described above, but without the RNA extraction step. As shown in Fig. 2, the one-step BVDV TaqMan assay successfully detected the virus in supernatants obtained from MDBK cells infected with BVDV1 (Fig. 2A), BVDV2 (Fig. 2B), and a mixture of BVDV1 and BVDV2 (Fig. 2C). A sample from uninfected MDBK cells (Fig. 2D) gave a negative result. When the same primers were used in a SYBR Green assay with RNA obtained from PI cattle, the virus was detected with a clear melting peak at approximately 80 °C, as shown in Supplementary Fig. S1.

### Cross-reactivity with BVDV3

When the BVD3 strain Italy-1/10-1 was tested using the one-step BVDV TaqMan assay, a negative result was obtained (Supplementary Fig. S2A). However, when the BVDV3 strains TH/04 KhonKaen and JS12/01 were tested, cross-reactivity was observed, and these two samples were incorrectly identified as BVDV-2 (Supplementary Fig. S2B and C). Sequence comparisons indicated that there was a mismatch in the sixth nucleotide from the 3’-terminal end of the BVDV2 probe that might have been responsible for these inconsistent results (Supplementary Table S3).

### Cross-reactivity with other pathogens

The cross-reactivity of the assay was evaluated by testing BVDV1 M6-1/G1B/Cattle/Japan/2018 and BVDV2 M2-1/G2A/Cattle/Japan/2016 as well as a collection of seven other viruses: BRSV, BCoV, AKAV, BoHV-1, BPIV-3, BIV, and BLV. We found that the TaqMan assay detected BVDV1 and BVDV2 but showed no cross-reactivity against any of the other seven viruses (Supplementary Fig. S3).

### Comparison of the LOD of the one-step BVDV TaqMan assay with other diagnostic methods

We determined the LOD of the one-step BVDV TaqMan assay using isolated RNA and compared the results with those obtained using the Hoffmann assay. The experiment was performed using serial tenfold dilutions (1, 10, 100, and 1000) of the RNA extracted from serum samples from BVDV1 (M5-3/G1B/Cattle/Japan/2018)- and BVDV2 (M2-2/G2A/Cattle/Japan/2018)-infected cattle, in triplicate. For BVDV1, the LOD was a 100-fold dilution, which is consistent with the Hoffmann assay. In the case of the BVDV2 genotype, our assay could detect a tenfold higher dilution than the Hoffmann assay, as shown in Table 2.

**Table 2:** Comparison of the LOD of viral RNA between the TaqMan assay and the WOAH reference assay (Hoffmann *et al.*, 2006)

	BVDV1 (OR674901)		BVDV2 (OR674907)	
	TaqMan assay	WOAH reference assay	TaqMan assay	WOAH reference assay
RNA dilution fold				
×1	3/3	3/3	3/3	3/3
×10	3/3	3/3	3/3	3/3
×100	3/3	3/3	3/3	0/3
×1000	0/3	0/3	0/3	0/3

Next, we compared the LOD between the direct TaqMan assay and VI, the gold standard method with the highest detection sensitivity. The LOD was determined by testing viral-titer-adjusted serum samples from cattle infected with BVDV1 (M6-1/G1B/Cattle/Japan/2018) or BVDV2 (M2-1/G2A/Cattle/Japan/2016) at 10^3^, 10^2^, 10^1^, and 10^0^ TCID_50_/mL, in triplicate. We found that the LOD of the direct TaqMan assay was similar to that of VI for both BVDV1 and BVDV2. However, VI could detect samples with a lower viral titer than the direct TaqMan assay (10^2^ TCID_50_/mL for BVDV1 and 10^1^ TCID_50_/mL for BVDV2) (Table 3).

**Table 3:** Comparison of the LOD of the direct TaqMan assay using serum samples without RNA isolation and that of virus isolation

	BVDV1 (OR674904)		BVDV2 (OR674894)	
	Direct TaqMan assay	Virus isolation	Direct TaqMan assay	Virus isolation
(TCID_50_/ml)				
10^3^	3/3	3/3	3/3	3/3
10^2^	2/3	3/3	3/3	3/3
10^1^	0/3	0/3	0/3	1/3
10^0^	0/3	0/3	0/3	0/3

### Correlation with virus titer and intra-assay reproducibility of the one-step BVDV TaqMan assay

The correlation between the direct TaqMan assay Ct values and the TCID_50_ was determined by testing serial dilutions (10^6^, 10^5^, 10^4^, 10^3^, 10^2^ TCID_50_/mL) of BVDV1 (M6-1/G1B/Cattle/Japan/2018) or BVDV2 (1/G2A/Cattle/Japan/2016). As shown in Supplementary Fig. S4, a strong correlation was found between the Ct value and the log_10_ TCID_50_ (R^2^ = 0.95 for BVDV1 and R^2^ = 0.99 for BVDV2). The average slope values for BVDV1 and BVDV2 were ˗3.32 and ˗3.69, respectively.

We also evaluated the intra-assay reproducibility of the one-step BVDV TaqMan assay. As shown in Table 4, the assay was highly reproducible and thus reliable, with the coefficient of variance ranging between 0.66% and 2.14% for BVDV1 and between 0.06% and 0.50% for BVDV2.

**Table 4:** Reproducibility of the direct TaqMan assay for detection of BVDV1 and BVDV2

	BVDV1 Intra-assay				BVDV2 Intra-assay			
	Mean Ct	±	SD	(CV%)	Mean Ct	±	SD	(CV%)
(TCID_50_/ml)								
10^6^	25.86	±	0.171	0.66	23.12	±	0.015	0.06
10^5^	29.77	±	0.357	1.19	27.83	±	0.141	0.50
10^4^	33.38	±	0.394	1.18	31.29	±	0.054	0.17
10^3^	37.12	±	0.794	2.14	35.05	±	0.110	0.31

SD, standard deviation; CV, coefficient of variation

### Agreement between results obtained with the one-step BVDV-genotyping TaqMan assay and phylogenetic analysis

To test the reliability of the assay for genotyping using bovine serum without RNA extraction, we tested sera from PI cattle from which the virus had already been phylogenetically characterized. The assay correctly identified the genotype of the virus in eight sera from cattle infected with BVDV1 regardless of subtype (A or B) and in four sera from cattle infected with BVDV2, without any observable cross-reactivity (Table 5).

**Table 5:** Correspondence between genotyping results using the TaqMan assay and phylogenetic analysis

Sample	Accession no. (5' UTR)	Subtype determined by phylogenetic analysis	TaqMan assay	
			BVDV1	BVDV2
1	ORF674893	1A	+	-
2	ORF674895	1B	+	-
3	ORF674896	1B	+	-
4	ORF674899	1B	+	-
5	ORF674901	1B	+	-
6	ORF674904	1B	+	-
7	ORF674912	1B	+	-
8	ORF674914	1B	+	-
9	ORF674894	2A	-	+
10	ORF674907	2A	-	+
11	ORF674908	2A	-	+
12	ORF674909	2A	-	+

## Discussion

The implementation of effective and early diagnostic testing procedures with high sensitivity and specificity is required as part of the global efforts to control and eradicate BVDV. To achieve this, we developed a one-step BVDV TaqMan assay that was able to detect and differentiate BVDV1 and BVDV2 genotypes directly, using bovine serum samples without RNA extraction. Previously, PCR-based diagnostics for BVDV required an RNA purification step [11, 13]. Our direct TaqMan assay can be used to diagnose BVDV infection with sensitivity and specificity equivalent to the previous methods, reducing the cost and time required for testing.

A two-step TaqMan assay [13] and a PCR-RFLP (restriction fragment length polymorphism) assay [22] for BVDV genotyping have been reported previously. In the two-step TaqMan assay, RNA extraction from samples and cDNA synthesis from RNA are performed prior to the amplification step, making the two-step assay more time- and labor-intensive than the one-step assay and increasing the chances of contamination, which can lead to false positive results and make it unsuitable for high-throughput diagnosis. PCR-RFLP requires an additional step of restriction enzyme digestion after RNA extraction and before PCR amplification, which is also time-consuming. Previously described one-step RT-PCR methods have required the extraction of RNA from numerous samples [11, 23–27], whereas our assay can be performed directly using serum samples in a single-tube reaction, making it cheaper, faster, and more suitable for high-throughput diagnosis.

The direct TaqMan assay reported here is highly specific for BVDV, showing no cross-reactivity with BRSV, BCoV, AKAV, BoHV-1, BPIV-3, BIV, or BLV, and is capable of detecting BVDV in serum samples with a viral titer as low as 10^3^ TCID_50_/mL for BVDV1 and 10^2^ TCID_50_/mL for BVDV2. For BVDV1, the detection limit was 10^2^ TCID_50_/mL in two out of three trials. Our assay showed either an equivalent or a lower LOD when compared with VI, which has the highest detection sensitivity among the established methods. However, VI is labor-intensive because it requires cell culture, sample inoculation, and immunostaining of infected cells, all of which together takes at least 3 days. Considering that the viral titer in sera from PI cattle varied from 10^2^ TCID_50_/mL to 10^6^ TCID_50_/mL [28], we predict that our assay will be able to detect the virus in most PI cattle.

The direct TaqMan assay has two main advantages that will make it suitable for detecting BVDV in cattle early in the infection process (Supplementary Table S4). First, it is capable of detecting the virus in serum without requiring RNA extraction. Second, it can be used to identify BVDV1 and BVDV2 in a single-tube reaction. These novel attributes confer advantages over previous techniques.

Recent studies have predominantly concentrated on evaluating the efficacy of BVDV vaccination in preventing fetal infection and reducing the incidence of persistent infection in calves [29]. Before 1995, most BVDV vaccines were produced using BVDV1 strains. More recently, however, there has been increasing use of modified-live and inactivated vaccines incorporating both BVDV1 and BVDV2 [30], since BVDV1 and BVDV2 are not cross-protective. In Germany, Ireland, and Scotland, vaccination has been used as a supplementary control tool to eradicate BVDV in these regions [31]. In this scenario, our assay may assist vaccine developers in identifying BVDV1 and BVDV2 strains in serum samples, facilitating informed decision-making regarding the formulation of combined vaccines.

On-site diagnosis, particularly in the case of newborn calves, is crucial for the timely identification of BVDV infection. Our assay offers promising prospects for on-site diagnosis, especially through the integration of portable PCR technology. Our assay is suitable for such applications because reactions are completed in a single tube without requiring RNA extraction.

One limitation of our assay is that it is not well suited for detection of BVDV3 strains. As shown in Supplementary Fig. S2, the BVDV3 strain Italy-1/10-1 strain was not detected by this assay. This is probably due to a mismatch in the center nucleotide of the BVDV2 probe, in the sixth position from the 3’ end. Currently, BVDV1 and BVDV2 are prevalent worldwide, whereas epidemics of BVDV3 have only been reported in a few countries, including the USA [32], China [33], and Italy [13]. Therefore, in countries where BVDV3 is prevalent, an additional test for BVDV3 is needed. The antigenicity of BVDV1, BVDV2, and BVDV3 is distinct, and vaccination against BVDV1 and BVDV2 does not protect against BVDV3 infection [34]. In the future, it should be possible to modify our assay so that it can also be used to detect BVDV3.

In conclusion, our newly developed direct TaqMan assay enables the rapid, efficient, and cost-effective detection and genotyping of BVDV1 and BVDV2. This new technique has potential applications in on-site diagnosis because it does not require RNA extraction and can be carried out in a single reaction tube. It may therefore contribute to the control and eradication of BVDV in the future.

## Supplementary Information

Below is the link to the electronic supplementary material.

Supplementary file1 (PDF 804 KB)

## Data Availability

No new data were created or analysed during this study. Data sharing is not applicable to this article.
