# Supplementary material for: Direct TaqMan assay for the detection and genotyping of bovine viral diarrhea virus types 1 and 2
Source: Arch Virol. 2024 Dec 12;170(1):15. doi: 10.1007/s00705-024-06207-z (PMC11638383; doi:10.1007/s00705-024-06207-z)
Supplement: Supplementary file 1 — Supplementary file1 (PDF 804 KB) [file 705_2024_6207_MOESM1_ESM.pdf]

Supplementary information

Direct TaqMan Assay for the Detection and Genotyping of Bovine Viral Diarrhea Virus Type 1 and 2

Shakir Ullah, Kosuke Notsu, Akatsuki Saito, Tamaki Okabayashi, Hirohisa Mekata, Norikazu Isoda, Satoshi Sekiguchi*

*Correspondence to: sekiguchi@cc.miyazaki-u.ac.jp

Contents

Figure S1: Performance of the BVDV-specific primer set in the SYBR Green assay.

Figure S2: Performance of designed primers and probes using the BVDV3 RNA fragment.

Figure S3: Specificity of the direct TaqMan assay.

Figure S4: Correlation between the viral titer and the TaqMan assay Ct value.

Table S1: BVDV strain information obtained from the NCBI database to determine conserved regions.

Table S2. The sequences of fragment-containing BVDV3 RNA.

Table S3. Sequence mismatches between primer/probe and tested BVDV3 RNA .

Table S4: List of comparison of different BVDV detection methods with Direct RT-qPCR.

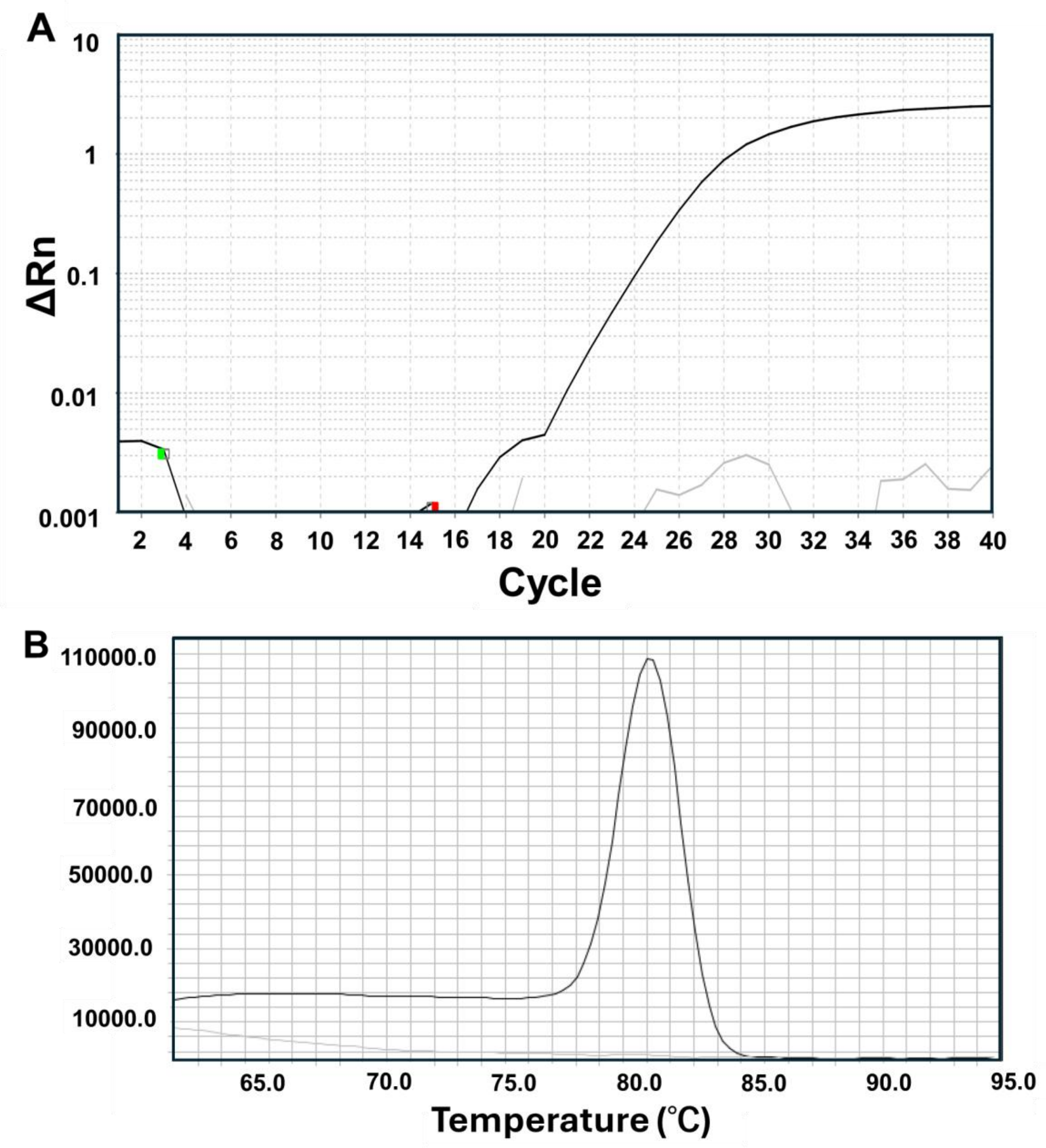

Fig. S1. Performance of the BVDV-specific primer set in the SYBR Green assay

BVDV-specific amplification using the SYBR Green assay is shown. BVDV positive and negative samples are indicated in black and gray, respectively. **(A)** The amplification plot of the SYBR Green assay. **(B)** The BVDV-positive sample showed a specific melting peak Tm at approximately 80 °C.

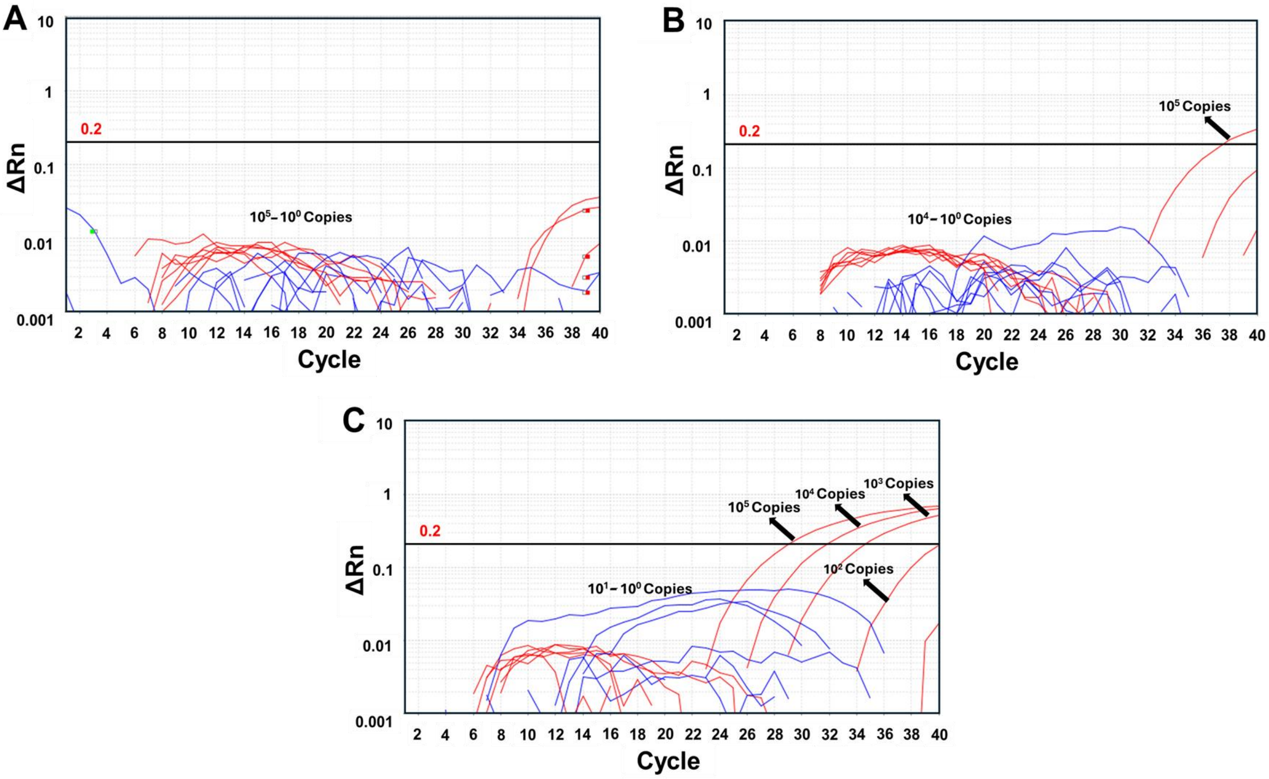

Figure S2: Performance of designed primers and probes using the BVDV3 RNA fragment.

Panels **(A)** to **(C)** show the amplification plots of the direct TaqMan assay for BVDV3 RNA . Amplification plots for the BVDV1 (FAM) and BVDV2 (HEX) channels are indicated in blue and red, respectively. **(A)** Italy-1/10-1 strain BVDV3 RNA **(B)** TH/04 KhonKaen strain BVDV3 RNA, and **(C)** JS12/01 strian BVDV3 RNA.

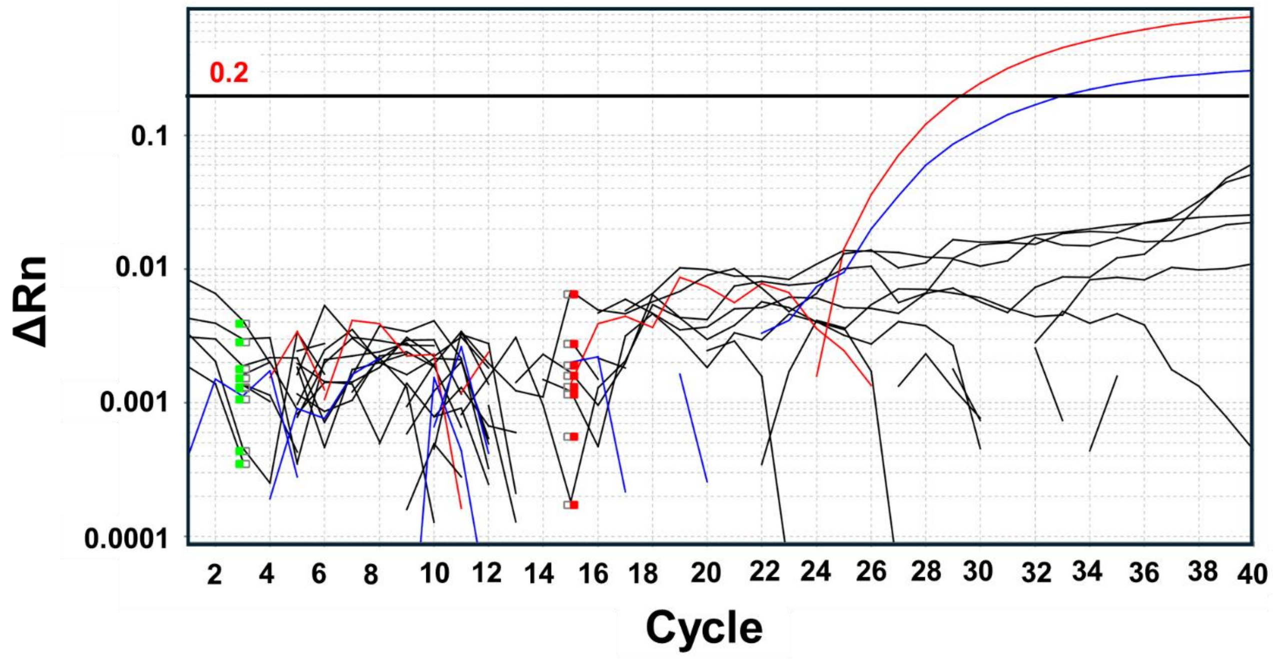

Fig. S3. Specificity of the direct TaqMan assay

Specificity of the direct TaqMan assay for BVDV1 and BVDV2 templates. Amplification plots for the BVDV1 (FAM) and BVDV2 (HEX) channels are indicated in blue and red, respectively. The dark gray amplification plots at the bottom represent different viruses including bovine respiratory syncytial virus (BRSV), bovine coronavirus (BCoV) strain CS5, Akabane virus (AKAV), bovine herpes virus 1 (BoHV-1), bovine parainfluenza virus 3 (BPIV-3), bovine immunodeficiency virus (BIV), and bovine leukemia virus.

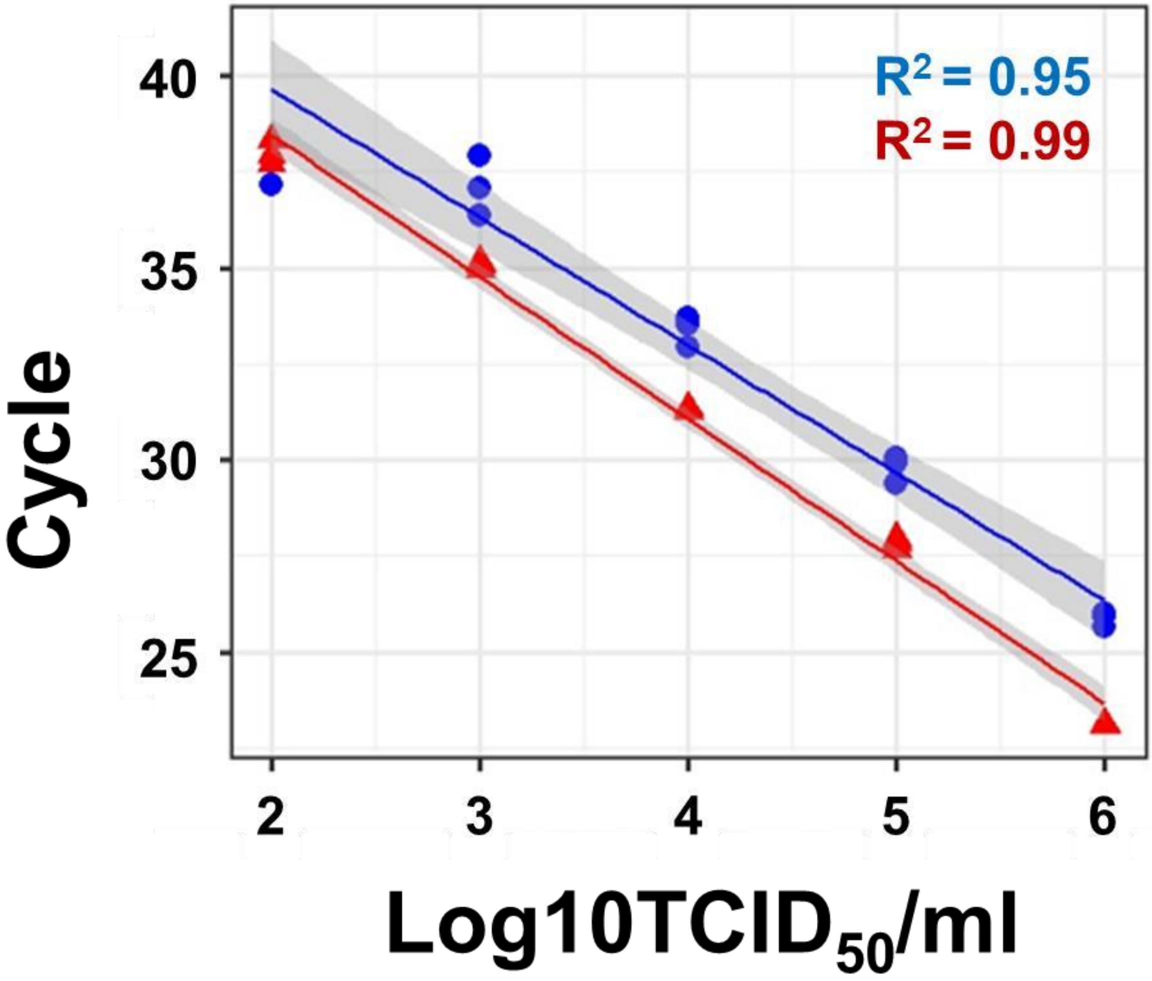

Fig. S4. Correlation between the viral titer and the TaqMan assay Ct value

The X axis indicates the log10 TCID50 and the Y axis indicates the Ct value. The blue plotted line shows BVDV1 values, and the red plotted line shows BVDV2 values. Each dot indicates the observed Ct in triplicates. The correlation between two variables is evaluated by linear regression analysis, as shown by the linear line.

Table S1. BVDV strain information obtained from the NCBI database to determine conserved regions.

Name of isolate	Subtype	Acc. No.	Name of isolate	Subtype	Acc. No.
Singer Arg	1a	DQ088995.2	CH-04-01b	1h	MW655625.1
Tokachi-22	1a	LC727647.1	SuwaCp	1k	KC853441.1
Tokachi-27	1a	LC727648.1	SD-15	1m	KR866116.1
1-NADL	1a	M31182.1	Shitara/02/06	1n	LC089876.1
CHN/HN	1a	ON901783.1	IS26/01ncp	1o	LC089875.1
Y2	1b	KY964311.1	camel 6	1q	KC695810.1
Tokachi-8	1b	LC727644.1	GBK_E+	2a	AB894423.1
Tokachi-13	1b	LC727645.1	SH2210-23	2a	HG426494.1
Tokachi-15	1b	LC727646.1	KZ-91-CP	2a	LC006970.1
Osloss	1b	M96687.1	Tokachi-47	2a	LC727649.1
Bega-like	1c	KF896608.1	Tokachi-31	2a	LC727650.1
10JJ-SKR	1d	KC757383.1	Olwein_*12	2a	MH231139.1
Isolate Carlito	1e	KP313732.1	Hokudai	2b	AB567658.1
SLO/1170/2000	1f	KX987157.1	Th/04_KhonKaen	3	FJ040215.1
UM/126/07	1h	LT631725.1	Italy-1/10-1	3	HQ231763.1

Table S2. The sequence of fragment-containing BVDV3 RNA.

Genotype	RNA sequence with Access number
BVDV3	>HQ231763_Italy-1/10-1_198-298<br>GUUCAAAGUUUAACAACUCCAUGUGCCAUGUACAGCAGAGUUUUUAUACUAGU<br>GGUGUAGCAAGUCUCUGCAACACCCUAUCAGGCUGUACUCCCAAAGAG
BVDV3	>FJ040215_Th/04 KhonKaen_305-405<br>GUUCAAAGUUUAACAACUCCAUGUGCCAUGUACAGCAGAGUUUUUAUACUAGU<br>GGUGUAGCAGGUCUCUGCAACACCCUAUCAGGCUGUACUCCCAAAGGC
BVDV3	> JX469119_JS12/01_233-333<br>GUUCAAAGUUUAACAAUUCCAUGUGCCAUGUACAGCAGAGUUUUUAUACUAGU<br>GAUGUAGCAGGUCUCUGCAGCACCCUAUCAGGCUGUACUCCCAAAGAG

Table S3. Sequence mismatches between primer/probe and tested BVDV3 RNA.

Sequence (5` to 3`)				
	Forward primer	BVDV1 Probe	BVDV2 Probe	Reverse primer
Primer/Probe	TRYGRAYACAGCCTGATAGGG	ACTR-AAAATCTCTGC	TGTAGCAGAGACCTGCTA	CTGTACATGGCACATGGAGTTGW
Italy-1/10-1	TGGGAGTACAGCCTGATAGGG	AGTATAAAAACTCTGC	TGTTGCAGAGACTTGCTA	CTGTACATGGCACATGGAGTTGT
Th/04_KhonKaen	TGGGAGTACAGCCTGATAGGG	AGTATAAAAACTCTGC	TGTTGCAGAGACCTGCTA	CTGTACATGGCACATGGARTTGT
JS12/01	TGGGAGTACAGCCTGATAGGG	AGTATAAAAACTCTGC	TGCTGCAGAGACCTGCTA	CTGTACATGGCACATGGAATTGT

Mismatched nucleotides with primer/probe sequence are indicated by red colour.

Table S4. List of comparison of different BVDV detection methods with Direct RT-qPCR

Name	PCR Steps	RNA extraction requirement	Genotyping BVDV	Reference
Ullah et al., 2024	1 step	No	Yes	Current study
Hoffmann B et al., 2006	1 step	Yes	No	[11]
Mari V et al., 2016	2 steps	Yes	Yes	[13]
Zhang J et al., 2022	1 step	Yes	No	[23]
Zhang YQ et al., 2015	1 step	Yes	Yes	[24]
Liang H et al., 2019	2 steps	Yes	No	[25]
Baxi M et al., 2006	1 step	Yes	Yes	[26]
Goto Y et al., 2021	2 steps	Yes	Yes	[27]
